# Reproducibility and Repeatability of Coronary Computed Tomography Angiography (CCTA) Image Segmentation in Detecting Atherosclerosis: A Radiomics Study

**DOI:** 10.3390/diagnostics12082007

**Published:** 2022-08-19

**Authors:** Mardhiyati Mohd Yunus, Akmal Sabarudin, Muhammad Khalis Abdul Karim, Puteri N. E. Nohuddin, Isa Azzaki Zainal, Mohd Shahril Mohd Shamsul, Ahmad Khairuddin Mohamed Yusof

**Affiliations:** 1 Programme of Diagnostic Imaging and Radiotherapy, Faculty of Health Sciences, Universiti Kebangsaan Malaysia (UKM), Kuala Lumpur 56000, Malaysia; 2 Programme of Medical Imaging, Faculty of Health Sciences, Universiti Selangor (UNISEL), Batang Berjuntai 40000, Selangor, Malaysia; 3 Department of Physics, Faculty of Science, Universiti Putra Malaysia (UPM), Serdang 43400, Selangor, Malaysia; 4 Institute of IR4.0, Universiti Kebangsaan Malaysia (UKM), Bangi 43600, Selangor, Malaysia; 5 Faculty of Business, Higher College of Technology, Sharjah P.O. Box 7947, United Arab Emirates; 6 Department of Radiology, Hospital Canselor Tunku Muhriz (HCTM), Kuala Lumpur 56000, Malaysia; 7 Imaging Centre, Institut Jantung Negara (IJN), Kuala Lumpur 50400, Malaysia

**Keywords:** atherosclerosis, CCTA, radiomics, repeatability, reproducibility

## Abstract

Atherosclerosis is known as the leading factor in heart disease with the highest mortality rate among the Malaysian population. Usually, the gold standard for diagnosing atherosclerosis is by using the coronary computed tomography angiography (CCTA) technique to look for plaque within the coronary artery. However, qualitative diagnosis for noncalcified atherosclerosis is vulnerable to false-positive diagnoses, as well as inconsistent reporting between observers. In this study, we assess the reproducibility and repeatability of segmenting atherosclerotic lesions manually and semiautomatically in CCTA images to identify the most appropriate CCTA image segmentation method for radiomics analysis to quantitatively extract the atherosclerotic lesion. Thirty (30) CCTA images were taken retrospectively from the radiology image database of Hospital Canselor Tuanku Muhriz (HCTM), Kuala Lumpur, Malaysia. We extract 11,700 radiomics features which include the first-order, second-order and shape features from 180 times of image segmentation. The interest vessels were segmentized manually and semiautomatically using LIFEx (Version 7.0.15, Institut Curie, Orsay, France) software by two independent radiology experts, focusing on three main coronary blood vessels. As a result, manual segmentation with a soft-tissuewindowing setting yielded higher repeatability as compared to semiautomatic segmentation with a significant intraclass correlation coefficient (intra-CC) 0.961 for thefirst-order and shape features; intra-CC of 0.924 for thesecond-order features with *p* < 0.001. Meanwhile, the semiautomatic segmentation has higher reproducibility as compared to manual segmentation with significant interclass correlation coefficient (inter-CC) of 0.920 (first-order features) and a good interclass correlation coefficient of 0.839 for the second-order features with *p* < 0.001. The first-order, shape order and second-order features for both manual and semiautomatic segmentation have an excellent percentage of reproducibility and repeatability (intra-CC > 0.9). In conclusion, semi-automated segmentation is recommended for inter-observer study while manual segmentation with soft tissue-windowing can be used for single observer study.

## 1. Introduction

According to a report from the World Health Organization, in 2019, ischemic heart disease (IHD) was the number one silent killer worldwide, including in Malaysia, with 17.9 million people dying every year due to cardiovascular disease (CVD) [1]. IHD occurs when there is a reduction in the oxygen content carried by the blood vessels to the heart. This can be caused by a blockage by plaque inside the wall area of the blood vessels, known as atherosclerosis. There are three types of atherosclerosis—calcified, semi calcified, and noncalcified plaque [2]. Generally, patients with suspected acute coronary syndrome (ACS) or even without symptoms can undergo the health-screening procedure of coronary computed tomography angiography (CCTA), the invasive coronary angiography (ICA) procedure, and intravascular ultrasound (IVUS) [3]. The radiologist will analyze the image and based on the percentage of blood vessel blockage, classify it as grades 0, 1, 2, 3, or 4 or use the standard reporting system in CCTA, which is CADRADS [4]. CADRADS was developed to standardize the reporting of CCTA, improve communication, and guide therapy. It was published in 2016 by the Society of Cardiovascular Computed Tomography (SCCT), the American College of Radiology (ACR), and the North American Society for Cardiovascular Imaging (NASCI) and has been endorsed by the American College of Cardiology (AC). CCTA has been acknowledged as a noninvasive technique to diagnose and detect atherosclerotic plaques [5,6]. CCTA can highlight the main vessels and identify the plaque morphology, enabling the planning of treatment and estimating the risk stratification in patients with CVD [5,6,7,8,9]. In clinical practice, the radiologist will visualize and interpret the CCTA by assessing the qualitative features of the images. However, this subjective evaluation requires full attention to detail, which may be overlooked if there is too much diagnostic study [10]. As the diagnosis is a subjective assessment highly dependent on the expertise level of the readers, interobserver reproducibility has been found to be low [4]. Hence, precision medicine was established, enabling more precise tools to detect and characterize diseases [11].

**Figure 1:**
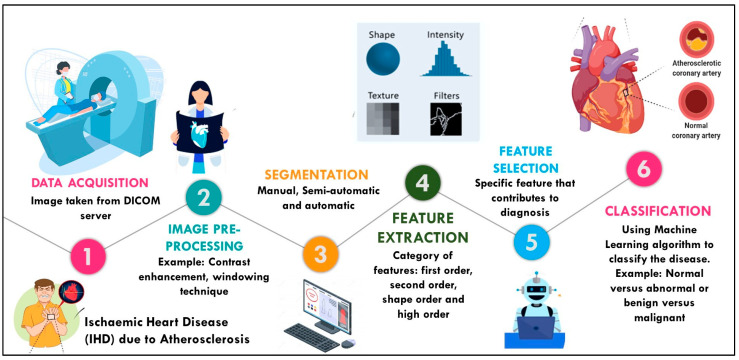
Radiomics Process.

In medical imaging, radiomics is a technique that extracts various features from medical images using data algorithms and usually represents them as tumor patterns and other disease characteristics [12,13]. The radiomics process starts with data acquisition, image preprocessing, image segmentation, feature extraction, feature selection, and image classification, as shown in Figure 1. In this research, we were focused more on the first four phases of radiomics study.

The use of radiomics in CCTA radiographs is considered vital as a diagnostic tool, due to its minimal requirements for acquisition and postprocessing [14]. It improves the characterization of plaque-containing blood vessels by using complex and potential algorithms to facilitate the radiologist’s decision regarding prospective patient treatment. The use of texture analysis or the so-called part of artificial intelligence (AI) technology is also a promising alternative quantitative analysis to identify and characterize the lesion by using medical imaging information such as from CCTA images [15,16,17,18,19]. Despite its ability, reproducibility in extracting radiomics features remains a concern.

Radiomics has an important role in machine-learning (ML) development and is regarded as the only high-throughput feature for medical images [20]. Radiomics is also subject to the limitation of visual image interpretation, as it covers most of the DICOM format images [21]. Previously, researchers found that data mining from radiographs and predictive analysis widened the scope of medical imaging [22,23,24,25]. This can enable prognostic models to classify the pattern and properties of the disease. However, according to Wang et al., the only obstacle to the use of the models is the reliability of their outcome [26].

Challenges during segmentation process of coronary artery in CCTA is inevitable, as the coverage area of the vessel is small and can cause variability in the segmentation result [27]. In addition, conventional manual segmentation is tedious and exhausting, which calls for faster and more reliable segmentation techniques. Previous works evaluated the semiautomatic segmentation methods and found their reliability similar to manual segmentation [28,29,30]. A work on mammography found that the flood-filling algorithm yields the best result, compared to the watershed and k-means algorithms [31]. Furthermore, the semiautomatic segmentation of the grow-cut algorithm on lung computed tomography (CT) was also found to have high reproducibility and robustness, compared to manual segmentation [32].

Feature extraction is essential to obtain relevant information on input images and represent that information in the lower-dimensionality space [33]. These features are extracted by using an advanced mathematical algorithm that describes phenotypes of lesions that might not be visible to the naked eye. Apart from the selection of segmentation algorithms, medical-image-segmentation accuracy will also be affected by other extrinsic factors such as image analysis, the observers’ educational background, image-segmentation-related experience, and the level of familiarity with the segmentation software [27,34]. To obtain accurate data regarding lesion characteristics, a robust image segmentation algorithm is required. Manual segmentation is widely used in clinical settings, especially for tumour contouring in the oncology sector and volume measurement in the radiology field. However, such segmentation performed through human observation and interpretation was shown to be susceptible to variation, apart from being time-consuming. Hence, this study aimed to evaluate the reproducibility and repeatability of manual and semiautomatic segmentation the atherosclerotic lesions in atherosclerosis of CCTA images. This work aims to identify the most appropriate CCTA image segmentation method for radiomics analysis to quantitatively extract the atherosclerotic lesion.

## 2. Materials and Methods

The CCTAs with contrast images (axial plane) were retrieved retrospectively from the picture archiving communicating system (PACS) system of Hospital Canselor Tuanku Muhriz (HCTM), Cheras, Kuala Lumpur, Malaysia. The images were collected randomly from December 2019 to March 2021. This observational clinical study was approved by the ethical-committee members on 4 January 2021 (vide approval No. UKM PPI/111/8/JEP-2020-751). Patient consent was waived due to the research involving no more than minimal risk to subjects. Thirty (30) CCTA images of coronary blood vessels (including calcified, noncalcified, and normal blood vessels) were selected using the rule of thumb in the reliability study [35]. The normal coronary artery was identified as a control group. Data were recorded based on random sampling, which is better in reducing bias and more random for optimal data collection results, including radiologist reports and patient demographic data.

**Figure 2:**
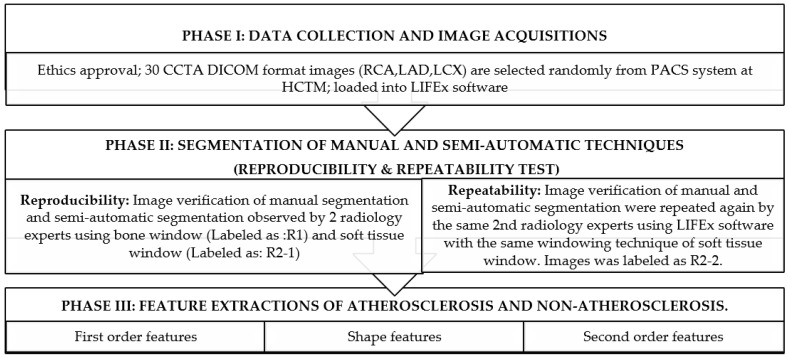
Overall research workflow.

This research used a single-blinded type of study whereby only the researcher knows the report of the patient. The report was used in the first place to distribute the patient selection based on patient diagnosis. The sample was taken randomly including normal patients, calcified plaque, and noncalcified plaque. Then, two radiology experts were blinded by not knowing the patient’s diagnosis and they were both not among those who carried out the reporting on the sample of images. Patient identifications were voided to reduce bias during the image segmentation process. The CCTA images were renamed into sequence of number to avoid patient identification being revealed during the segmentation process. Both manual and semiautomatic segmentation processes were carried out using Local Image Feature Extraction (LIFEx) software (version 7.0.16, Orsay, France) [36]. LIFEx is a free and open-source software platform for analyzing 3-dimensional (3D) medical images, intended for research purposes only, and has not been reviewed or approved by the Food and Drug Administration (FDA) for clinical purposes. Figure 2 shows the overall research workflow in comparing and analyzing the radiomics features that were extracted.

The inclusion criteria included contrast enhanced CCTA with atherosclerosis confirmed by radiologists based on the radiologist report. The exclusion criteria were coronary artery bypass graft (CABG) patients or patients under treatment, stenting, or ballooning, as well as poor-quality CCTA images (with artefacts) and extremely obese patients (body mass index (BMI) more than 35). All the patients were scanned using 320-Multidetector Computed Tomography (CT) Toshiba Aquilion One (Canon Medical Systems, Tochigi, Japan) with a standard prospective electrocardiogram (ECG) protocol. The protocols were pre-set with a tube voltage of 120 kVp, a tube current of 600 mA with automatic mA modulation technique enabled, a gantry rotation time of 350 ms with half gantry turn (half segment reconstruction), a collimation aperture of 0.5 mm × 160, and 0.537 mm-thickness slices with an overlap distance of 0.5 mm. Ensuring a noise index of 10, the scan size area was 16 cm^2^ to allow the entire heart image to be scanned in one gantry rotation in one heartbeat (dynamic volume scan). The scanning range was set between a *Z*-axis of 120 cm and 160 cm, starting at the bifurcation branch of the trachea until the diaphragm. Throughout the scan, the ECG detection technique was set at 70–80% at the peak of the R-R interval (the interval between one heartbeat and another) [37]. In addition, no table movement per gantry round (pitch) was used because the scan mode used was sequential (step-and-shoot). There was no table movement and overlap of scans during the radiation emitted.

Radiomics feature extractions for manual and semiautomatic segmentation were compared and analyzed. To measure the reproducibility of both (manual and semiautomatic) segmentations, two different methods of viewing bone windows (labeled as: R1; window width (WW): 2500; window level (WL): 480) and soft-tissue windows (labeled as R2-1: window width (WW): 450–1000; window level (WL): 100–300) by two radiology experts were carried out. Then, the manual and semiautomatic segmentations were repeated by the 2nd radiology expert so that we could measure the repeatability using the intraclass correlation (ICC). We labelled it as R2-2 (soft-tissue window setting; window width (WW): 450–1000; window level (WL): 100–300). Both radiologists viewed the coronary blood vessels and segmentized the image relating to the areas of calcified atherosclerosis, noncalcified atherosclerosis, and normal vessels independently. The soft-tissue- and bone-windowing techniques were used by each radiology expert accordingly based on the windowing technique that they commonly used for viewing the CCTA images in their radiology reporting workstation.

**Figure 3:**
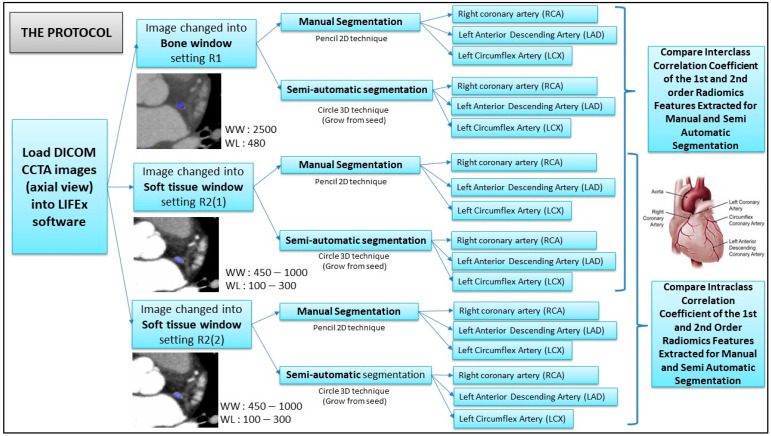
The framework of the research protocol.

Based on the lesion (volume of interest: VOI) areas of the CCTA images on the axial plane, only 3 axial slices of images were chosen to be segmentized. This is to standardize the VOI techniques of segmentation area on each vessel. The LIFEx software extracted the features of atherosclerosis and non-atherosclerosis using a radiomics application into first-order features, shape order features and secondorder features. The interclass and intraclass correlation (ICC) was counted as poor (ICC < 0.5), moderate (0.5 < ICC < 0.75), good (0.75 < ICC < 0.9), and excellent (ICC > 0.9), as referred to in [35]. The details of the research workflow are presented in Figure 3 below.

### 2.1. Manual Segmentation (Pencil 2D Technique) Protocol

The loading axial plane of the CCTA data images through the digital imaging and communications in medicine (DICOM) module was loaded into the LIFEx software. Patient identification was removed to reduce any bias-related factors. The observation was conducted under a good room light setting in a private room. Technically, a Windows 10 (64-bit) GPU unit of Intel^®^ Core i7 processor with 16GB RAM was used to run these images. Soon after, the observers identified the location of atherosclerotic plaques and non-atherosclerotic plaques. The DICOM axial images were adjusted with bone window setting (window width (WW): 2500; window level (WL): 480) for radiologist R1. The soft-tissue window setting was set to (WW: 450–1000; WL: 100–300), and was adjusted for radiologist R2-1 and R2-2 (second attempt) independently.

**Figure 4:**
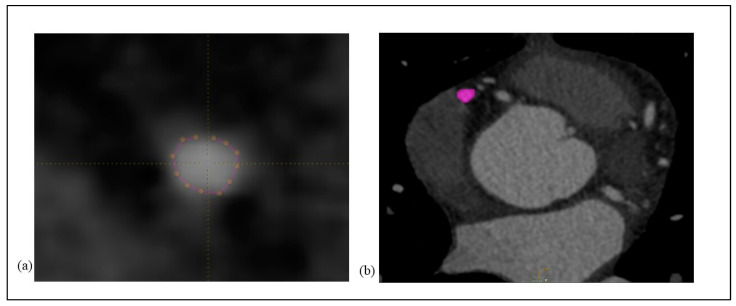
LIFEx software was used to perform manual and semiautomatic segmentation on RCA, LAD, and LCX. (**a**) Manual segmentation using pencil 2D technique with soft-tissue-windowing setting on RCA (**b**) The area of segmentation covered after manual segmentation with soft-tissue-windowing setting was performed on RCA.

Next, the image was zoomed to 10 times enlargement to focus more on the coronary blood vessel. The pencil 2D technique (manual segmentation) was selected. The nodes were added around the lesion region surrounding the coronary artery using the mouse cursor. The region of interest was then colored in purple (as shown in Figure 4) once the nodes were connected circularly to the first node. This step was repeatedly carried out on two more slices (three slices of axial CCTA in total). The same technique was implemented on two more vessels of coronary arteries.

### 2.2. Semiautomatic Segmentation (Circle 3D Technique) Protocol

**Figure 5:**
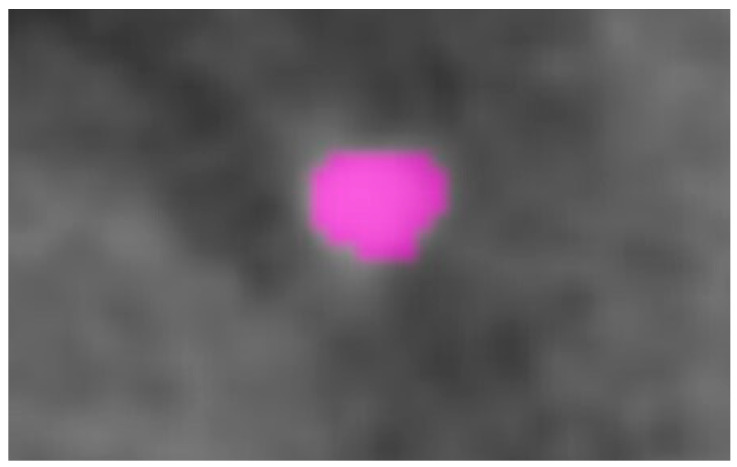
Semiautomatic segmentation using circle 3D technique in LIFEx software in bone window setting on the coronary blood vessel.

The same technique was repeated using semiautomatic (circle 3D technique also known as growth from seed) segmentation. The chosen nodes were able to adjust the size according to the circular shape of the coronary blood vessel size. The nodes were added around the lesion region surrounding the coronary artery, using the mouse cursor. Subsequently, the flood fill effects were activated, and VOI segmented according to similar voxels’ intensity (as shown in Figure 5). To finalize the output, the segmented lesion was manually edited either to delete some extra covered area or to add an area not covered by the semiautomatic cursor in finalizing the semiautomatic segmentation.

The flood-filled algorithm is capable of connecting the area in the multidimensional array by allowing the related intensity voxels to access the selected node, which is decided by the users. This algorithm is comparable with the bucket tool in paint programs, which fills connected, similar-intensity voxels with a different color [31]. The algorithm was initiated with the first node during the selection of the volume of interest (VOI). The pixels were connected directionally from the first node to the former. Subsequently, the intensity voxels were decided, and the algorithm detected the identified path and switched to a different color. To reduce the leakage effect, the VOI was manipulated using the neighborhood size parameter.

### 2.3. Feature Extraction

Once the segmentation process was finished, the feature was extracted using the textural extraction analysis in the LIFEx software, with a bin size of 10. All features were extracted based on the mathematical algorithm predicated on pixel intensities. The first-order, shape order, and second (2nd)-order features were extracted to a Microsoft Excel worksheet (CSV. formatted file). Of each of the 30 images, we repeated 6 times the image segmentation where each technique produced three types of radiomics features which were shape, texture, and lesion intensity. We managed to extract 11,700 radiomic features of the CCTA images to allow quantification of the atherosclerotic and non-atherosclerotic lesion characteristics. Table 1 summarizes the composition of features which are divided into three groups: (I) intensity (1st-order features), (II) shape order features, and (III) textural (2nd-order features). The total features extracted from the volume of interest for lesion intensity, shape, and texture were 29, 5, and 31, respectively.

The first-order statistics could distinguish the histogram of voxel intensity within the atherosclerotic lesion region on CCTA images. Furthermore, the shape features were able to describe the volume properties of the lesion. The patterns or spatial distributions of voxel intensities derived from the gray-level dependence matrix (GLDM), gray-level co-occurrence matrix (GLCM), and gray-level run-length matrices (GLRLM) were defined by the textural features. The features from the co-occurrence and run-length matrices were estimated by averaging all the 13 symmetric directions in three dimensions [38].

Several interest features, such as entropy, contrast, uniformity, and correlation, are presented in the equation below:

(1) $$\mathrm{Entropy}=-\sum_{i=1}^{N_{g}}p\left(i\right)\log_{2}\left(p\left(i\right)+\varepsilon \right)$$

Entropy measures the average amount of information required to encode the image values, where $N_{g}$ = the number of non-zero bins and p(i) = normalized first-order histogram.

(2) $$\mathrm{Contrast}=\sum_{i=1}^{N_{g}}\sum_{j=1}^{N_{g}}{\left(i-j\right)}^{2}p\left(i,j\right)$$

Contrast determines the local intensity variation present in the image. A larger value correlates with the greater disparity in intensity values among neighboring voxels.

(3) $$\mathrm{Uniformity}=\sum_{i=1}^{N_{g}}p{\left(i\right)}^{2}$$

Uniformity measures the sum of squares of each intensity value. A greater uniformity implies greater homogeneity. Correlation defines the linear dependency of gray-level values to their respective voxels in GLCM. The value for correlation is between 0 (uncorrelated) and 1 (perfectly correlated). The equation of correlation is shown below:

(4) $$\mathrm{Correlation}=\frac{\sum_{i=1}^{N_{g}}\sum_{j=1}^{N_{g}}p\left(i,j\right)\mathrm{ij}-\mu_{x}\mu_{y}}{\sigma_{x}\left(i\right)\sigma_{y}\left(j\right)}$$

where $\mu_{x}$, $\mu_{y}$ and $\sigma_{x}$, $\sigma_{y}$ are mean gray-level intensity and standard deviation of p_x_ and p_y_*,* respectively. Table 2 summarizes the radiomic characteristics derived in this investigation in detail.

### 2.4. Statistical Analysis

The intraclass correlation coefficient (intra-CC) represents the correlations within the class of data and is used to determine the repeatability of the extracted features, while the reproducibility, which was determined by the interclass correlation (inter-CC) analysis, was used in this study. Briefly, the relationship between the observer was measured and categorized into three models which can be chosen appropriately, depending on the experimental situation. In this study, variance values were estimated to define the interobserver segmentations by using a two-way mixed-effect model of analysis of variance (ANOVA) [24]. Mathematically, ICC is defined as:

(5) $$\mathrm{ICC}\;\left(A,1\right)=\frac{{\mathrm{MS}}_{R}-{\mathrm{MS}}_{E}}{{\mathrm{MS}}_{R}+\left(k+1\right){\mathrm{MS}}_{g}+\frac{k}{n}\left({\mathrm{MS}}_{C}-{\mathrm{MS}}_{E}\right)}$$

One-way analysis of variance (ANOVA) was used to obtain the ICC values for intra observer segmentation [14,15]. The equation below defines ICC (C,1):

(6) $$\mathrm{ICC}\left(C,1\right)=\frac{{\mathrm{MS}}_{R}-{\mathrm{MS}}_{W}}{{\mathrm{MS}}_{R}+\left(k-1\right){\mathrm{MS}}_{W}}$$

where MS_R_ = mean square for rows, MS_W_ = mean square for residual sources of variance, MS_E_ = mean square error, MS_C_ = mean square for columns, and k and n are the numbers of observers involved and subjects.

The reproducibility evaluation was performed by letting one observer segmentize 30 images 2 weeks apart. The measurement was based on multiple initializations of the segmentation algorithm from the same observer. Interobserver reproducibility was determined from several observers according to the segmentation technique and the degree of agreement between observers. The Wilcoxon rank-sum test with a *p*-value set at 0.05 was used to define the differences in reproducibility. We used the Statistical Package for Social Sciences (SPSS, also known as IBM SPSS statistics) version 25 (SPSS, Chicago, IL, USA) to analyze the data and all data were expressed in (mean ± standard deviation).

## 3. Results

### 3.1. Descriptive Analysis

Of 30 images of CCTA, 50% were categorized as coronary artery disease—reporting and data system (CADRADS)-4, 30% as CADRADS 2, 10% as CADRADS 1, and 10% as CADRADS 5. In total, 36.7% were calcified atherosclerosis, 23.3% were noncalcified plaque, and 40% were normal.

### 3.2. ICC for Manual and Semiautomatic Segmentation for First (1st)-Order and Shape Order Features

**Figure 6:**
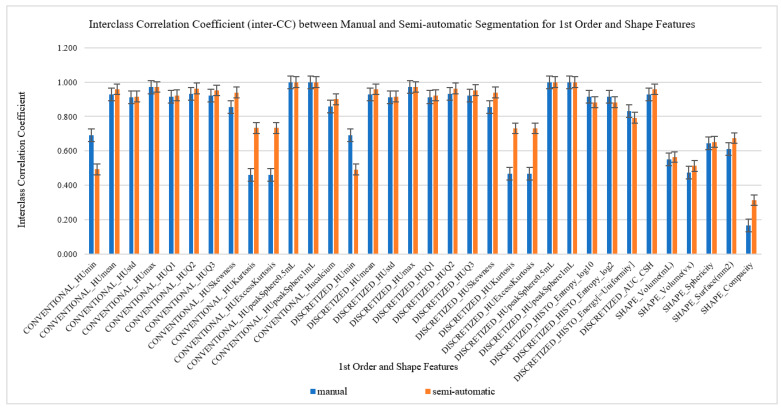
Interclass Correlation Coefficients (inter-CC) between manual and semi-automatic segmentation for 1st order features including the shape features to compare its reproducibility.

**Figure 7:**
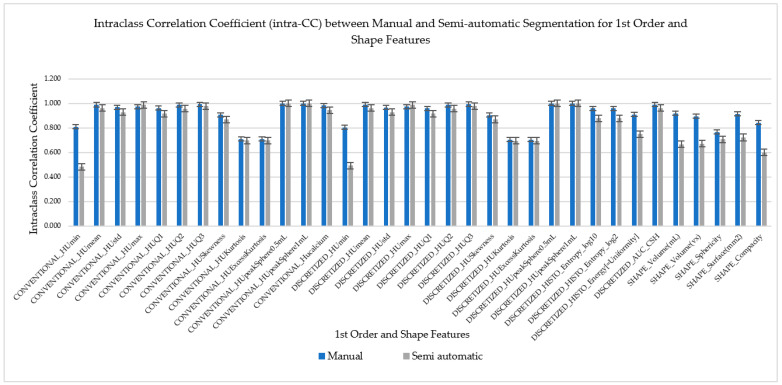
Intraclass Correlation Coefficient (intra-CC) between manual and semi-automatic segmentation for 1st order Features including the Shape Features to compare its repeatability.

Both inter- and intra class coefficients (ICC) for manual and semi-automatic segmentation of the first-order and shape order radiomics features were compared by the means and standard deviation, as shown in Figure 6 and Figure 7. The first 29 radiomics features were categorized as the first-order features followed by five types of shape order features. These radiomics features were then compared to their ICC to find their reproducibility and repeatability on both types of segmentation which were manual and semiautomatic. As we can visually analyze from the pattern of the bar chart graphs, we can see that the majority (52.9% (18/34)) of semiautomatic segmentation was higher in reproducibility as compared to manual segmentation, while the majority of the first-order and shape order features showed higher repeatability on manual segmentation (82.4% (28/34)).

### 3.3. ICC for Manual and Semiautomatic Segmentation for Second-Order Features

**Figure 8:**
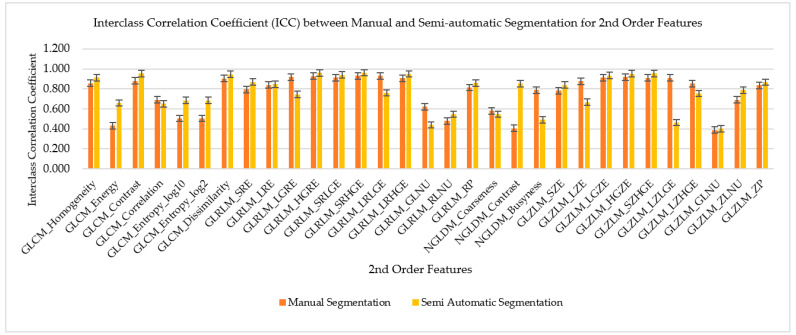
Interclass Correlation Coefficient (inter-CC) between manual and semi-automatic segmentation for 2nd order features to compare its reproducibility.

**Figure 9:**
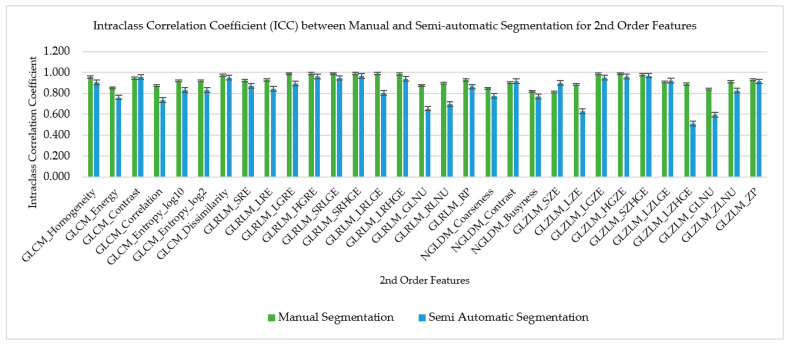
Intraclass Correlation Coefficient (inter-CC) between manual and semi-automatic segmentation for 2nd order features to compare its repeatability.

The ICC for manual and semiautomatic segmentation of the second-order radiomics features was compared by the means and standard deviation as shown in Figure 8 and Figure 9. There were 31 radiomics features of the second order which were extracted from the CCTA images. These radiomics features were then compared to their ICC to find their reproducibility and repeatability on both types of segmentation which were manual and semiautomatic. The bar chart graphs show the majority (71% (22/31)) of semiautomatic segmentation was higher in reproducibility as compared to manual segmentation, while the majority of the second-order radiomic features showed higher repeatability on manual segmentation (83.9% (26/31)).

**Figure 10:**
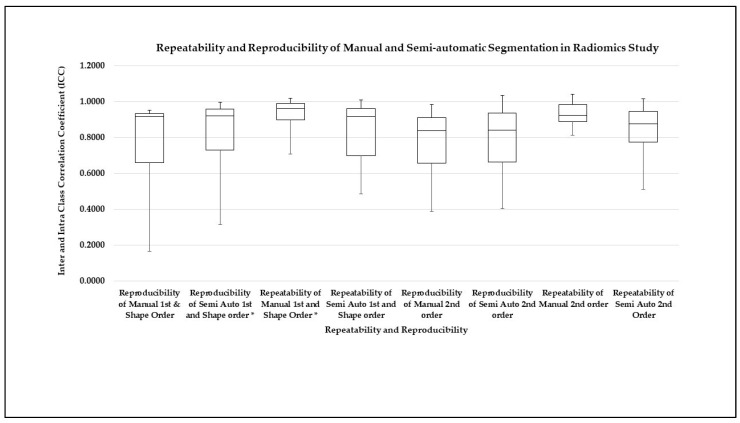
Repeatability and reproducibility of manual and semi-automatic segmentation for the first-, shape and second-order features. The (*) shows the highest repeatability and reproducibility mean score with a significant *p*-value (*p* < 0.001).

Figure 10 shows the overall result in a boxplot graph of comparing the reproducibility and repeatability between manual and semiautomatic segmentation for all radiomics features. It is clearly shown that the highest repeatability was in manual segmentation for the first-order and shape order features with an excellent median of ICC = 0.961 (*p* < 0.001), and ICC = 0.924 (*p* < 0.001) for the second-order features. The average mean of repeatability and reproducibility for the first-order and shape features’ (Cronbach’s alpha) measured ICC was 0.979 (*p* < 0.001) and 0.859 (*p* < 0.001) for the second-order features. Meanwhile, the highest reproducibility in semiautomatic segmentation for the first-order and shape order features had an excellent median of ICC 0.920 (*p* < 0.001).

### 3.4. ICC Level on Reproducibility and Repeatability for Manual and Semiautomatic Segmentation

We categorized both ICCs as poor (ICC < 0.5), moderate (0.5 < ICC < 0.75), good (0.75 < ICC < 0.9), or excellent (ICC > 0.9) [35]. Table 3 shows the reproducibility and repeatability of manual and semi-automatic segmentation for the first-order, shape order, and second-order features. Consequently, semiautomatic segmentation had the highest percentage of excellent (ICC > 0.9) reproducibility for the segmentation of coronary blood vessels with atherosclerotic lesions and normal blood vessels for first-order features. Meanwhile, manual segmentation had the highest percentage of excellent (ICC > 0.9) reproducibility for second-order features. Meanwhile, manual segmentation also gained the highest percentage of (ICC > 0.9) repeatability for segmentation with 74% for first-order features and 68% (excellent ICC > 0.9) for second-order features. Comparably, the manual and semiautomatic segmentations both yielded a majority of excellent (ICC > 0.9) repeatability and reproducibility based on the first-order, shape order, and second-order features.

## 4. Discussion

This study shows that manual segmentation yielded a high repeatability result since the same observer was using the same method and windowing technique during the image segmentation process. This result shows that using a soft-tissue-windowing setting in viewing the CCTA images for image segmentation purposes was able to produce high repeatability. Yet, there are still some disadvantages to the manual technique which is monotonous, time-consuming, and did affect the interobserver variability [39] as shown in the result above.

Meanwhile, semi-automatic segmentation is proven to have higher reproducibility in CCTA images segmentation, using two different windowing techniques. Other than higher reproducibility result, semi-automatic segmentation also taking less time in segmentation as compared to the manual segmentation [39]. This result also shows that the windowing technique also gave impact on reproducing the same segmentation result in manual segmentation. It is suggested to maintain a standard image viewing setting as it may affect the image visualization, especially in detecting atherosclerosis. This result is supported by the previous study conducted on other imaging modalities, diseases, and techniques of segmentation [40].

Although semiautomated segmentation was proved less susceptible to intra- and interobserver variability compared to manual delineation [41,42], it may still cause contrasting results even using the same segmentation approach. This may be due to the requirement of human interaction in semiautomated segmentation; different initialization points’ locations and sizes may cause different outcomes [43]. This was also supported by Owen in 2013 [44] who found that with less human interaction during the segmentation process, a more reproducible result could be obtained. Other than that, they suggested using the same segmentation software and approach in obtaining radiomics information because significant inter-software variability was observed in their study. The same suggestion was also mentioned in theother study [45].

The advantages of semiautomated segmentation include allowing the viewer to detect those areas of interest which have about the same pixel value beside it. This has been proven beneficial to apply, especially in detecting disease in small areas [34]. This will be one of the reasons why semi-automated segmentation has lower repeatability as compared to manual segmentation. 

Notably, the majority of the first-order, shape order and second-order features for both manual and semiautomatic segmentation had excellent reproducibility and repeatability with (ICC > 0.9). Yet, the average percentage of excellent level of reproducibility and repeatability (ICC > 0.9) was higher in the first-order and shape order features. This is due to the technical approach of measuring the first-order and second-order features itself, where the first-order features only focus on the mathematical calculation of the Hounsfield unit in one single pixel, while the second-order features are prone to measure the pixel next to another pixel of the image to be measured. Yet, these radiomics features will be highly beneficial in supervising various automated machine-learning models, especially in detecting any diseases in medical images particularly at the small area. This result is supported by the previous study [15,16,46].

The segmentation process is very crucial in radiomics study. If the area of segmentation is carried out wrongly, it may affect the machine-learning codes to classify diseases from the CCTA images. Therefore, this research is very prominent to enhance the importance of choosing the best segmentation technique in comparing with two different windowing techniques in CT scan. The data that we managed to extract out from the CCTA images were consisted of large number of patients’ diagnostic informations which will be very helpful in predicting the disease quantitatively by usingproper image windowing and segmentationtechnique. 

Meanwhile, this research simulated the segmentation techniques in two different centers in Malaysia. With reference to this study, it is hoped that future research can apply the semi-automated segmentation in soft-tissue windowing setting accordingly. A previous study also was conducted on CT lung images as a reference [47]. This may help radiologists to refer to the standard guideline in viewing the CCTA images, especially in image segmentation of coronary blood vessels. This will give a large contribution to patient management, hospital management, and finally, to the nation in detecting atherosclerosis. It is suggested for future research to further analyze the radiomics features of two different segmentation techniques by measuring the accuracy of image classification in detecting atherosclerosis using a machine-learning algorithm. Therefore, it may give a higher impact in terms of the accuracy in detecting atherosclerosis before implementation to a real prospective patient.

The limitation of this study is that it involved only two radiology experts from two different centers. It can also be improved in the next reliability research study by adding a minimum of three radiologists to interpret the images [35]. Yet, this research is highly impactful in studying the method and protocol that can be implemented in a future study.

## 5. Conclusions

This study demonstrated the manual radiomics feature extraction with soft-tissue-windowing setting yielded higher repeatability as compared to semiautomatic segmentation, with 82.4% for first-order/shape Features and 83.9% for second-order features. Meanwhile, semiautomatic segmentation had higher reproducibility as compared to manual segmentation, with 71% for first-order/shape features and 52.9% for second-order features. The majority of the first-order and second-order features for both manual and semiautomatic segmentation had an excellent repeatability and reproducibility (ICC > 0.9). In conclusion, semiautomated segmentation is recommended for interobserver study while manual segmentation with soft-tissue windowing can be used for single-observer study.

## Figures and Tables

**Table 1:** Composition of 11,700 radiomic features extracted using LIFEx software.

Features (*n* = 180)	Radiomics Features
Lesion intensity (1st-order features)	29 × 180
Shape order features	5 × 180
Texture (2nd-order features)	31 × 180

**Table 2:** List of first-order features (*n* = 29), second-order features (*n* = 31), and shape features (*n* = 5). Reprinted/adapted with permission from Ref. [15].

First-Order Features (*n* = 29)	Second-Order Features (*n* = 31)	Shape Order Features (*n* = 5)
**Conventional:** CONVENTIONAL_min CONVENTIONAL_mean CONVENTIONAL_std CONVENTIONAL_max CONVENTIONAL_Q1 CONVENTIONAL_Q2 CONVENTIONAL_Q3 CONVENTIONAL_Skewness CONVENTIONAL_Kurtosis CONVENTIONAL_Excess_Kurtosis CONVENTIONAL_peak_Sphere_0.5mL CONVENTIONAL_peak_Sphere_1mL CONVENTIONAL_calcium_AgatstonScore **Discretized:** DISCRETIZED_min DISCRETIZED_mean DISCRETIZED_std DISCRETIZED_max DISCRETIZED_Q1 DISCRETIZED_Q2 DISCRETIZED_Q3 DISCRETIZED_Skewness DISCRETIZED_Kurtosis DISCRETIZED_ExcessKurtosis DISCRETIZED_peakSphere0.5 mL DISCRETIZED_peakSphere1 mL DISCRETIZED_HISTO_Entropy_log^10^ DISCRETIZED_HISTO_Entropy_log^2^ DISCRETIZED_HISTO_Energy DISCRETIZED_AUC_CSH	**Gray-Level Co-Occurrence Matrix (GLCM):** GLCM_Homogeneity GLCM_Energy GLCM_Contrast GLCM_Correlation GLCM_Entropy_log^10^ GLCM_Entropy_log^2^ GLCM_Dissimilarity **Gray-Level Run Length Matrix (GLRLM):** GLRLM_Short Run Emphasis (SRE) GLRLM_Long Run Emphasis (LRE) GLRLM_Low Gray Run Emphasis (LGRE) GLRLM_High Gray Run Emphasis (HGRE) GLRLM_hort Run Low Gray-Level Emphasis (SRLGE) GLRLM_Short Run High Gray-Level Emphasis (SRHGE) GLRLM_Long Run Low Gray-Level Emphasis (LRLGE) GLRLM_Long Run High Gray-Level Emphasis (LRHGE) GLRLM_GLNU (Gray-Level Non-Uniformity) GLRLM_Run-Length Non-Uniformity (RLNU) GLRLM_Run Percentage (RP) **Neighborhood Gray-Level Differences Matrix (NGLDM):** NGLDM_Coarseness NGLDM_Contrast NGLDM_Busyness **Gray-Level Zone Length Matrix (GLZLM):** GLZLM_Short Zone Emphasis (SZE) GLZLM_Long Zone Emphasis (LZE) GLZLM_Low Gray-level Zone Emphasis (LGZE) GLZLM_High Gray-level Zone Emphasis (HGZE) GLZLM_Short Zone High Gray-Level Emphasize (SZHGE) GLZLM_Long Zone Low Gray-Level Emphasize (LZLGE) GLZLM_Long Zone High Gray-Level Emphasize (LZHGE) GLZLM_Gray-Level Non-Uniformity (GLNU) GLZLM_Zone-Length Non-Uniformity (ZLNU) GLZLM_Zone Percentage (ZP)	**Shape Features:** SHAPE Volume (mL) SHAPE_Volume (vx) SHAPE_Sphericity SHAPE_Surface (mm^2^) SHAPE_Compacity

**Table 3:** Comparison of reproducibility and repeatability of Manual and Semiautomatic Segmentation based on ICC levels (* shows the highest % for excellent ICC).

Radiomics Features	ICC Level	Type of ICC	Manual	Semi-Automatic
First-order and shape order	Excellent (ICC > 0.9)	Reproducibility (inter-CC)	19 (56%)	20 (59%) *
		Repeatability (intra-CC)	25 (74%) *	18 (53%)
	Good (0.75 < ICC < 0.9)	Reproducibility (inter-CC)	4 (12%)	7 (21%)
		Repeatability (intra-CC)	5 (15%)	5 (15%)
	Moderate (0.5 < ICC < 0.75)	Reproducibility (inter-CC)	5 (15%)	4 (12%)
		Repeatability (intra-CC)	4 (12%)	9 (26%)
	Low (ICC < 0.5)	Reproducibility (inter-CC)	6 (18%)	3 (9%)
		Repeatability (intra-CC)	0 (0%)	2 (6%)
Second order	Excellent (ICC > 0.9)	Reproducibility (inter-CC)	11 (35%) *	10 (32%)
		Repeatability (intra-CC)	21 (68%) *	13 (42%)
	Good (0.75 < ICC < 0.9)	Reproducibility (inter-CC)	10 (32%)	9 (29%)
		Repeatability (intra-CC)	10 (32%)	12 (39%)
	Moderate (0.5 < ICC < 0.75)	Reproducibility (inter-CC)	6 (19%)	8 (26%)
		Repeatability (intra-CC)	0 (0%)	6 (19%)
	Low (ICC < 0.5)	Reproducibility (inter-CC)	4 (13%)	4 (13%)
		Repeatability (intra-CC)	0 (0%)	0 (0%)

## Data Availability

Not applicable.
